# Recent Advances and Applications of Holographic 3D Display

**DOI:** 10.34133/research.1274

**Published:** 2026-05-07

**Authors:** Qian Huang, Rui-Yi Zhao, Chen-Liang Chang, Yi-Wei Zheng, Yi-Long Li, Zhao-Song Li, Nan-Nan Li, Chao Liu, Da-Wei Zhang, Song-Lin Zhuang, Di Wang

**Affiliations:** ^1^ School of Instrumentation and Optoelectronic Engineering, Beihang University, Beijing 100191, China.; ^2^ International Terahertz Research Center, Hangzhou International Innovation Institute, Beihang University, Hangzhou 311115, China.; ^3^ School of Optical-Electrical and Computer Engineering, University of Shanghai for Science and Technology, Shanghai 200093, China.; ^4^ Institute of Machinery Manufacturing Technology, China Academy of Engineering Physics, Mianyang 621900, China.; ^5^ School of Electronics and Information, Zhengzhou University of Light Industry, Zhengzhou 450002, China.

## Abstract

Due to the importance of vision in human lives, 3-dimensional (3D) display has become a key and hot topic in the field of information research. With the advancement of computer science technology, holographic 3D display, capable of fully recording and reconstructing the complete wavefront information of 3D scenes, is considered as one of the most ideal 3D display technologies and has long been pursued by researchers. In recent years, substantial breakthroughs have been achieved in holographic 3D display; however, some bottlenecks continue to constrain its applications, such as slow hologram generation speed and limited space–bandwidth product. Here, the technical bottlenecks restricting the further development of holographic display are comprehensively analyzed. The latest development and progress of the key technologies of holographic 3D display are summarized. First, different fast generation methods for computer-generated holograms are analyzed. Then, the latest progress of holographic 3D display with high space–bandwidth product and low speckle noise is summarized. Finally, various application fields of holographic 3D display technology and its future development trends are discussed. This is aimed to summarize and analyze the key issues and progress in holographic 3D display technology and gain a deeper understanding of the cutting-edge developments in this rapidly developing field, providing assistance for its future applications in holographic near-eye display, virtual reality, augmented reality, optical encryption, and other fields.

## Introduction

Approximately 70% to 80% of the information obtained by humans originates from vision, making visual display one of the most important ways for modern information delivery. Traditional 2-dimensional (2D) display technology is unable to provide depth information, limiting the immersive visual experience for viewers. In contrast, 3D display technology can produce virtual 3D objects in the real world, which presents a stereoscopic effect and provides viewers with a more immersive viewing experience. However, there is vergence-accommodation conflict in traditional 3D display based on binocular parallax principle, which will lead to dizziness and discomfort for viewers during prolonged viewing. As an emerging display technology, holographic 3D display technology is undergoing vigorous development in the world. Holography enables the recording and reconstruction of the complete wavefront information of 3D objects, as well as electromagnetic wave field with higher frequencies [1,2]. Therefore, it can faithfully reconstruct the 3D object in space, which lets the viewer directly observe the reconstructed 3D object, effectively resolving the vergence-accommodation conflict issue. For this vital discovery, Dennis Gabor was awarded the Nobel Prize in Physics.

After years of research, holographic 3D display technology has emerged as a pillar to promote the development across a wide range of optical and nonoptical technologies. It demonstrates significant application potential in diverse fields such as medical diagnosis, defense and security, aerospace, industrial manufacturing, education, and entertainment [3–7]. For instance, when it is applied to x-ray imaging, holographic 3D display technology enables the simultaneous reconstruction of volumetric images of bones and organs [8]. In data storage, holographic 3D display technology allows a huge amount of information to be stored in an extremely small area [9]. The combination of holographic technology and microscope systems can achieve precise observation of microscopic biological cells, providing a wide range of applications in fields such as medicine and biology [10]. In addition, with the rapid development of the micro/nanofabrication technologies, metasurface-based holographic 3D display also offers great advantages in the fields of anticounterfeiting and encryption [11].

Holographic 3D display technology has been greatly advanced over recent years, but there are still some problems and challenges that continue to restrict its development. (a) Slow calculation speed, hindering real-time display [12]. Due to the vast amount of information inherent of 3D objects, the calculation speed and refresh speed of existing holograms are difficult to achieve 3D display with real-time performance. (b) Limited viewing angle, falling to meet viewing requirements [13]. The wavelength of visible light is usually in nanometer level, whereas the pixel size of existing diffraction modulation device is typically in micrometer level, which causes a mismatch and leads to a small viewing angle with only a few degrees. (c) Severe speckle noise, degrading display quality [14]. Due to the high coherence of the laser source and the introduction of random phase, reconstructed images are susceptible to speckle noise and cross-talk, which significantly compromises their fidelity. Consequently, existing holographic 3D display still faces challenges in commercial applications. In recent years, researchers have done a lot of research on addressing the above problems, and the combination of different disciplines and technologies has also greatly improved the holographic 3D display effect.

In this paper, the latest frontier progress in holographic 3D display technology during recent years is organized, the present situation of holographic 3D display is classified and expounded, and the future development is prospected. Regarding the key technologies of holographic 3D display, the fast hologram generation technologies are first summarized, and different approaches of holographic 3D display with large information capacity and low speckle noise are then analyzed. Finally, the different application fields and developments of holographic 3D display are summarized.

## Key Technologies in Holographic 3D Display

**Fig. 1.**
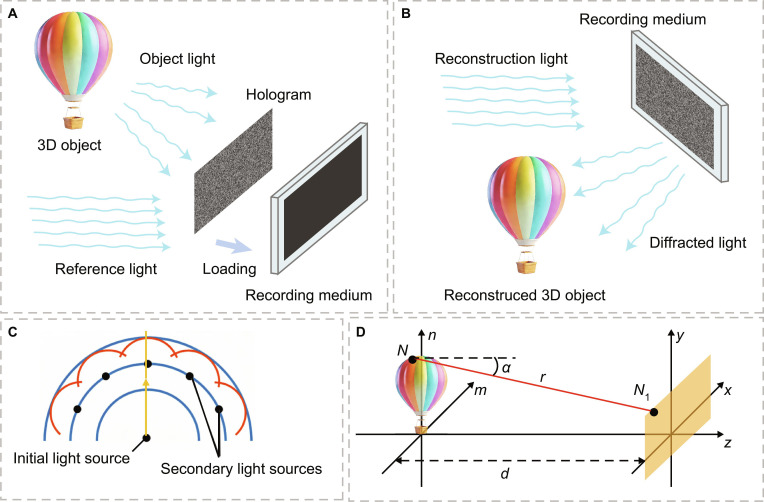
Principle of holographic 3-dimensional (3D) display technology. (A) Hologram generation and (B) holographic reconstruction processes of holographic 3D display. (C) Huygens’ principle. (D) Scalar diffraction diagram of point objects.

Holographic 3D display technology is mainly divided into 2 stages: hologram generation and holographic reconstruction, as shown in Fig. 1A and B. In the hologram generation process, based on the principle of optical interference, the wavefront information contained in object light of a 3D object is recorded by using a reference light, and the interference pattern is obtained as the hologram, which is then be loaded on to the recording medium. Subsequently, in the holographic reconstruction process, by illuminating the hologram with the reconstruction light, the 3D object can be fully reconstructed in space based on the principle of optical diffraction.

During the hologram generation process, the diffraction propagation of the object wave from the 3D object to the hologram plane needs to be calculated. This process is typically approximated on the basis of scalar diffraction theory. In 1960, Huygens proposed that each point on a wavefront can be regarded as a source of secondary spherical wavelets, and at any subsequent moment, the envelope of these wavelets forms the new wavefront, as shown in Fig. 1C. Later, Fresnel extended Huygens’ theory by suggesting that the light emitted from the same source should be coherent. Therefore, any point outside the wavefront is the result of coherent superposition of all secondary wavelets [15]. When a beam of light passes through an aperture plane *Σ*, at any observation point *M* behind the aperture, there exists a point *M*_1_ on the aperture plane that acts as a secondary wavelet source, emitting spherical wavelets. According to the Huygens–Fresnel principle, the optical field at point *M* can be expressed as:

$$\begin{array}{c} U\left(M\right)=\frac{1}{\mathrm{i\lambda}}\iint_{\Sigma }U\left(M_{1}\right)\frac{\text{exp}\left({\mathrm{ikr}}_{0}\right)}{r_{0}}\text{cos}\left(\theta \right)ds, \end{array}$$ (1)

where *r*_0_ represents the distance from *M*_1_ to *M*, *θ* represents the angle between the propagation direction and the normal vector, *i* is the imaginary unit, *λ* is the wavelength, *k* is the wavenumber, and *U*(*M*) and *U*(*M*_1_) are the optical field distributions at points *M* and *M*_1_, respectively.

Considering that the diffraction process from an object point *N* on the 3D object to the hologram plane plays a key role in hologram generation, as shown in Fig. 1D. The object point *N* lies on the object plane (*m*, *n*), while the diffraction point *N*_1_ lies on the hologram plane (*x*, *y*). The distance between these 2 points is set to *r*, the angle between the propagation direction and the optical axis *z* is set to *α*, and the distance from the object plane to the hologram plane is set to *d*. Based on the geometric relationship, the optical field distribution at point *N*_1_ can be obtained by:

$$\begin{array}{c} U\left(N_{1}\right)=\frac{1}{\mathrm{i\lambda}}\iint_{\Sigma }U\left(N\right)\frac{\text{exp}\left(\mathrm{ikr}\right)}{r^{2}}dmdn, \end{array}$$ (2)

where *U*(*N*) and *U*(*N*_1_) are the optical field distribution at points *N* and *N*_1_, respectively. By using the paraxial approximation, *U*(*N*_1_) can be expressed as:

UN1=1iλdexpikdexpik2dx2+y2.∬ΣUNexpik2dm2+n2exp−ikdxm+yndmdn (3)

By using the Fresnel approximation, the complex amplitude distribution on the hologram plane corresponding to each object point can be calculated, and the final complex amplitude distribution *U*(*x*, *y*) on the hologram plane of the 3D object can be obtained by superimposing all such contributions:

$$\begin{array}{c} U\left(x,\;y\right)=\sum_{j=1}^{P}\frac{A_{j}}{\sqrt{{\left(x-x_{j}\right)}^{2}+{\left(y-y_{j}\right)}^{2}+z_{j}^{2}}}\text{exp}\\ \left[i\left(k\sqrt{{\left(x-x_{j}\right)}^{2}+{\left(y-y_{j}\right)}^{2}+z_{j}^{2}}+\phi_{j}\right)\right], \end{array}$$ (4)

where *A_j_* and *φ_j_* represent the amplitude distribution and random phase distribution of the *j*th point, respectively. Supposing that there are *P* point sources on a 3D object and the resolution of the hologram is *a* × *b*, it is necessary to generate a single hologram of size *a* × *b* for total *P* times, with each time involving numerous multiplication and integral operations, which severely limits the generation speed.

Furthermore, current holographic 3D display technologies mostly rely on spatial light modulators (SLMs) for holographic reconstruction. However, SLMs typically support phase-only or amplitude-only modulation, with phase-only modulation being more common. Therefore, after obtaining the complex amplitude distribution on the hologram plane, the phase information needs to be extracted for holographic reconstruction. This extraction not only increases the calculation burden but also results in information loss, thereby affecting the reconstruction quality. These are critical issues that need to be addressed in the development of holographic 3D display technology.

### Fast generation technology of the computer-generated hologram

The fast generation of computer-generated holograms (CGHs) represents a key challenge in holographic 3D display technology. For a 3D object, generating the CGH primarily involves simulation calculations of the diffraction propagation from the 3D object to the hologram plane. When the recorded 3D object is highly complex with large size, the amount of information that needs to be calculated is enormous, leading to a significantly reduction in calculation speed. Therefore, accelerating the generation speed of CGH is important [16,17].

In general, the fast generation technology of CGHs can be achieved from algorithm improvement and hardware acceleration. Among them, algorithm improvement can fundamentally refine the calculation strategy of CGHs and reduce the amount of data that needs to be processed. Based on different mathematical analysis methods for 3D objects, CGH generation algorithm can be classified into 3 main categories, including point-based method, polygon-based method, and layer-based method [18].

#### Point-based method

The point-based method models a 3D object as a series of discrete point light sources [19]. The corresponding CGH is generated by calculating and superimposing the interference pattern of spherical wave emitted by each point onto the holographic plane. Due to the fact that each object point needs to participate in individual diffraction calculations, the calculation cost is extremely high.

**Fig. 2.**
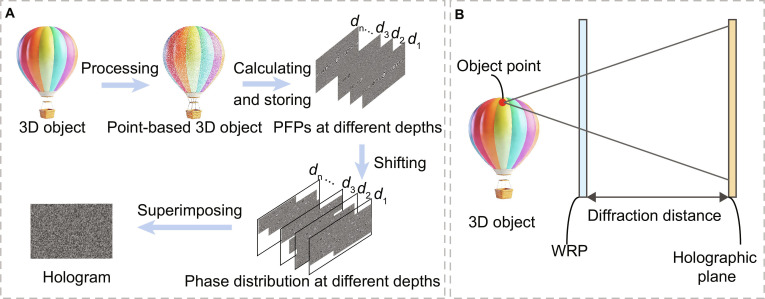
Point-based methods for fast computer-generated hologram (CGH) generation. (A) Process of the novel look-up table (NLUT) method. (B) Principle of the wavefront recording plane (WRP) method.

To accelerate the CGH generation speed with point-based method, researchers have proposed 2 key approaches: the look-up table (LUT) method and the wavefront recording plane (WRP) method. The LUT method precalculates and stores all fringe patterns (FPs) corresponding to each point of a 3D object [20]. During the CGH generation process, the relevant FP for each object point is simply retrieved from the table and superimposed. Although the LUT method offers high calculation efficiency, it requires substantial storage space because the FP of each object point needs to be prestored, which is a major challenge for computers. To reduce the storage requirement, Kim and Kim [21] proposed a novel LUT (NLUT) method. The flowchart of NLUT method is shown in Fig. 2A. In this approach, object points are grouped by depth information, and only the principal FPs (PFPs) of the center point for each depth plane are precalculated and stored. Compared with the LUT method, this method greatly reduces the number of precalculated patterns, thereby saving significant storage space while achieving a speedup of nearly 744 times.

Furthermore, by increasing the total number of shifted pixels in PFPs, researchers proposed an improved NLUT method, which achieved ~30% increase in the CGH generation speed while maintaining the resolution of 3D objects [22,23]. In addition, the required storage space was significantly reduced from megabytes to kilobytes. However, the number of PFPs required in the NLUT method is proportional to the depth layers of the 3D object. As depth layers increase, both the calculation complexity and storage requirements increase significantly. To address this problem, Pan et al. [24] proposed a split LUT method that converted CGH calculation into multiplication of two 1D datasets, reducing storage space while increasing calculation speed by approximately 700 times compared to the LUT method. Jia et al. [25] proposed a compressed LUT method, which required precalculating the PFP for only one depth plane and introduced the horizontal and vertical light modulation factors to generate holograms for different depth planes through multiplication.

In fact, the CGH generation process involves redundant calculations that do not contribute to the final reconstruction. To address this, an effective viewing area analysis method was proposed [26]. The size of the effective viewing area was related to the resolution of the 3D object, the positions of the object points, the parameters of the SLM, and the reconstruction distance. By only calculating the diffraction within the effective viewing area on the holographic plane, the storage space required for PFPs was greatly reduced, and the calculation time was improved by 10% compared to the NLUT method. In recent years, researchers have further optimized the effective regions involved in CGH generation to achieve acceleration for point-based method [27–29].

Unlike the LUT method, the WRP method manages storage space more effectively by introducing an intermediate WRP between the 3D object and the holographic plane [30]. As shown in Fig. 2B, the spherical waves emitted from a 3D object point are first recorded on the WRP, and then the wavefront is propagated from the WRP to the holographic plane by using the fast Fourier transform. According to geometric relationships, the closer the WRP is to the 3D object, the smaller the required recording area is, thereby reducing the calculation complexity and the storage requirement. To further accelerate CGH generation, Shimobaba et al. [31] incorporated LUT method to calculate the patterns at the WRP and then used graphics processing unit (GPU) to accelerate the diffraction propagation from the WRP to the holographic plane. This approach increased the CGH generation speed by 80 times compared to the conventional WRP method. However, the calculation efficiency of the WRP method is influenced by the size and depth range of the 3D object [32]. To overcome these limitations, researchers have developed different improved approaches, including WRP method based on shifted Fresnel diffraction and dual- and multi-WRP-based methods [33–35].

Given that CGHs generated by the point-based methods can accurately reconstruct detailed information of 3D objects, researchers continue to explore further optimizations to accelerate the generation process [36,37]. For instance, Villa-Hernández et al. [38] used the symmetry of object geometric distribution to accelerate the generation of Fresnel–Kirchhoff CGH, which preserved the high-precision advantage of point-based method and was especially suitable in metrology and precise wavefront reconstruction.

#### Polygon-based method

**Fig. 3.**
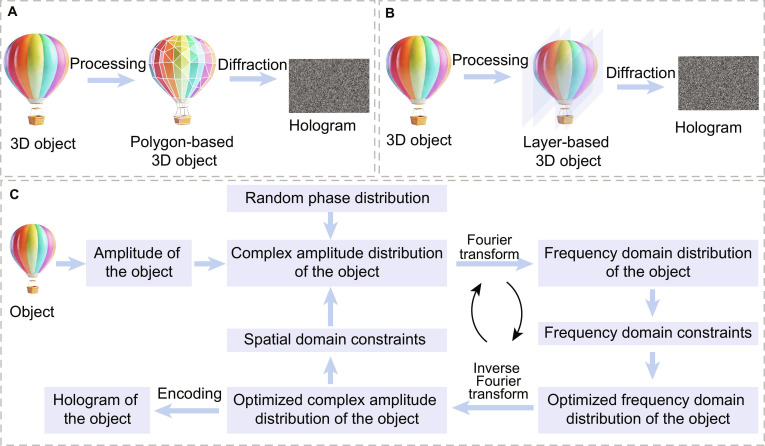
Polygon-based and layer-based methods for fast computer-generated hologram (CGH) generation. (A) Diagram of the polygon-based method. (B) Diagram of the layer-based method. (C) Flowchart of the Gerchberg–Saxton (GS) algorithm.

As shown in Fig. 3A, based on the surface features of 3D objects, the polygon-based method describes a 3D object as a series of polygons, and each polygon is regarded as a light source [39]. Compared to the point-based methods, this method can reduce the calculation cost by 2 to 3 orders of magnitude. According to different sampling strategies, polygon-based methods can be classified into traditional polygon-based method and analytical polygon-based method.

The traditional polygon-based method requires performing Fourier transform operations in both the frequency and spatial domains for each polygon, and it can reconstruct 3D objects with high realism by rendering each polygon individually. In 1988, Leseberg and Frère [40] proposed using the Fresnel approximation to calculate diffraction patterns on tilted planes. After adding phase information to the complex amplitude distribution of each polygon, a Fourier transform was performed, and the coordinate transformations in the frequency domain were used to map of each polygon onto the holographic plane. To improve the calculation efficiency, Pan et al. [41] proposed a fast polygon-based 3D affine transformation method grounded in 2D Fourier analysis. Starting from an original polygon, the diffracted light field of any other polygon in the 3D object was derived through pseudo-inverse matrix operation, interpolation, and power spectral density compensation. This method eliminated the additional diffusion calculations and overcame the high calculation cost of traditional polygon-based method, thereby reducing the calculation time by ~25%.

Furthermore, Ahrenberg et al. [42] proposed the analytical polygon-based method, which leveraged the Fourier transform analytical expression for isosceles right triangle and used affine transformations to establish the relationship between arbitrary triangles and the isosceles right triangle. Combined with the angular spectrum propagation on the rotating plane, the spectral distribution of the polygon on the holographic plane can be obtained. Compared to traditional polygon-based methods, the analytical polygon-based method only requires a single Fourier transform operation for 3D objects composed of multiple polygons, thereby greatly improving the CGH generation speed. In 2024, Wang et al. [43] proposed an improved rapid continuous shading method based on a fully analytical polygon algorithm. By combining square and triangular segments, they achieved nearly double the computational efficiency. In 2025, Nishi and Matsushima [44] rendered transparent objects using a polygon-based algorithm combined with wave optics, significantly reducing the computational load associated with wavefront propagation.

#### Layer-based method

The layer-based method decomposes a 3D object into a series of 2D slices parallel to the holographic plane according to its depth information, as shown in Fig. 3B [45]. Each slice processed independently contains the amplitude and phase information of the 3D object at a specific depth, and the final CGH corresponding to the 3D object is obtained by superimposing the contributions from all slices. Consequently, compared to point-based and polygon-based methods, the layer-based method demonstrates lower calculation complexity and greater flexibility in application.

In 2015, Zhao et al. [46] proposed a layer-based method based on angular spectrum diffraction. In this method, the amplitude information of each layer was overlaid with a random phase and propagated through angular spectrum diffraction. The spectral distribution of the 3D object was obtained by overlaying the distributions of all layers in the holographic plane, after which the complex amplitude distribution of the CGH was obtained via an inverse Fourier transform. Importantly, for a certain number of layers, the calculation complexity of this method was independent of the complexity of 3D objects, enabling CGH generation at speeds over 100 times faster than the conventional point-based methods. To further accelerate layer-based methods, Jia et al. [47] proposed a fast 2-step layer-based method by using subsparse 2D fast Fourier transform. Considering the occlusion effect between different layers, each layer contained a large area with zero-valued pixels, and only the nonzero areas of each layer were calculated. Moreover, the subsparse 2D fast Fourier transform operated solely on the rows and columns of nonzero pixels, which reduced calculation complexity and increased CGH generation speed by at least 3 times. In 2022, Yasuki et al. [48] reduced time-consuming complex operations using real-valued Fourier transform and Hartley transform, which increased the calculation speed by 3 times compared to conventional methods while maintaining comparable reconstruction quality.

Indeed, although layer-based methods improve the CGH generation speed, the calculation time remains proportional to the number of depth layers. In 2024, a fast CGH generation method based on the split Lohmann lens diffraction model was proposed, which achieved high-quality CGH in a single-step diffraction calculation, without relying on the number of layers [49]. This approach ran tens of times faster than traditional layer-based methods and could generate CGHs within milliseconds even on ordinary central processing units (such as Intel i5-1035G4), offering a promising way for real-time dynamic holographic 3D display.

In conclusion, each of the above 3 fast generation technologies of CGH has its own advantages. The point-based method requires precise diffraction calculation for each object point, resulting in extremely high calculation complexity, slow generation speed, and large storage requirements. The point-based method preserves detailed information of each object point, making it suitable for high fidelity reconstruction and for sparse point cloud patterns. The polygon-based method groups object points based on the surface features of the 3D object, achieving lower calculation complexity than the point-based method, significantly improving CGH generation speed, and greatly reducing storage requirements. Moreover, because the polygon-based method preserves the geometric characteristics of object surfaces, it is better suited for CGH generation of complex geometries with continuous surfaces and for 3D scenes requiring surface texture rendering. In addition, the layer-based method performs fast diffraction calculation for each depth layer. Compared with point-based and polygon-based methods, it can offer a lower calculation complexity and storage requirement.

#### Iterative method

As an important category of hologram generation technologies, iterative method has undergone decades of development. In 1972, Gerchberg and Saxton [50] proposed an iterative algorithm for CGH based on Fourier transform, which was called Gerchberg–Saxton (GS) algorithm. The flowchart of the GS algorithm is shown in Fig. 3C. By alternately applying the Fourier transform and inverse Fourier transform, GS algorithm iterates the complex amplitude distribution of the object between the spatial and frequency domains while imposing constraints in both domains, ultimately generating a CGH. However, although the iterative approaches cyclically optimize the phase distribution of the CGH, the iteration process itself is time-consuming. In 2017, Zhang et al. [51] developed the nonconvex optimization for volumetric algorithm, which employs a limited memory quasi-Newton gradient descent method to achieve rapid convergence of the phase distribution. This method not only improves the efficiency of iterative CGH generation but also significantly reduces memory consumption.

In 2019, Chakravarthula et al. [52] further extended the nonconvex optimization problem for holographic phase retrieval based on Wirtinger derivatives. In 2021, Chen et al. [53] proposed the stochastic gradient descent method, which used a loss function based on complex amplitude distribution rather than the traditional one based on the amplitude distribution. As a result, this method requires only a single evaluation of the complex amplitude during loss calculation, significantly reducing the CGH generation time for iterative algorithms. With the advancement of computer technology, the speed of iterative algorithms continues to improve, providing an important solution for the rapid generation of high-quality CGHs.

#### Hardware acceleration method

In recent years, with the rapid advancement of hardware platform computational power, researchers have utilized high performance hardware devices to accelerate CGH generation. Conventional CGH generation methods predominantly rely on central processing units for processing, which lack the ability to perform parallel data processing, seriously limiting the calculation efficiency. In contrast, GPUs not only provide parallel computing capabilities but also support programming with advanced programming languages, which greatly facilitate the debugging process during development [54]. In 2021, Lee et al. [55] proposed an out-of-core GPU algorithm that overcomes GPU memory limitations for large-scale hologram generation. The proposed method reduced the generation time of high-resolution holograms by 28%. In 2025, Lagrange et al. [56] proposed a one-step algorithm based on the split Lohmann method to efficiently generate CGH. By implementing on a GPU, the proposed method can increase the generation speed by 18.1 times compared with the traditional layer-based method.

Furthermore, GPUs are used for deep-learning-based methods. In 2018, Horisaki et al. [57] applied deep neural network to holography and proposed a CGH generation method by using residual convolutional neural networks (CNNs). In this method, the calculation time for a single CGH was only 26 ms. To achieve faster generation of large size CGH, Eybposh et al. [58] developed a deep neural network for generating CGH. By using reconstructed multilayer images and original multilayer 3D objects for error comparison and network updating, this method achieved a single CGH with resolutions of 1,024 × 1,024 within 10 ms. Peng et al. [59] proposed a neural holographic framework based on camera-in-the-loop training, which achieved a major breakthrough in speed, capable of generating full-color CGHs at approximately 40 fps on a GPU while maintaining a high image quality with peak signal-to-noise ratio ≈ 30 dB, striking a notable balance between high fidelity and real-time performance. Furthermore, by stochastically sampling wavelengths and angles and using only a few samples per iteration for gradient computation, Peng et al. [60] proposed a wave propagation model suitable for partially coherent light sources, significantly reducing the computational load of the model and enables efficient hologram optimization.

**Fig. 4.**
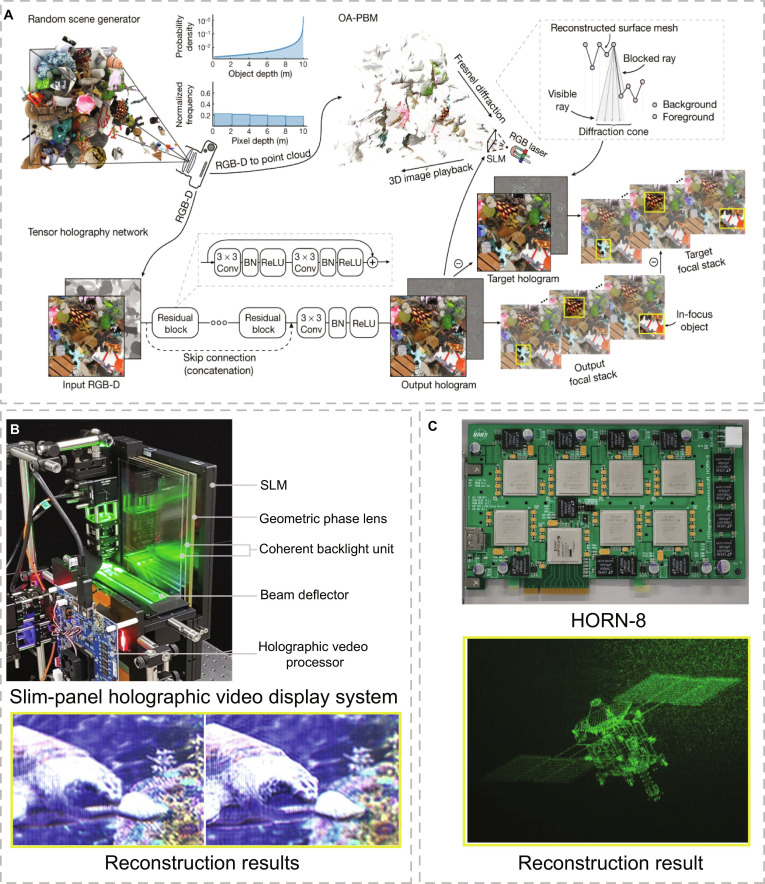
Hardware acceleration methods for fast computer-generated hologram (CGH) generation. (A) Structure of the network of Ref. [62]. RGB-D, red–green–blue-depth; BN, batch normalization; ReLU, rectified linear unit. (B) Prototype of the slim-panel holographic video display system and the reconstruction results [65]. (C) Prototype of the HORN-8 and the reconstruction result [73]. References [62,65,73] are reprinted with permission from Springer Nature.

In addition, in 2021, Wu et al. [61] used U-Net as the holoencoder to generate CGH and used Fresnel propagation model as the decoder to reconstruct the images. This approach enabled the generation of a single CGH with resolutions of 3,840 × 2,160 within 150 ms. Shi et al. [62] synthesized high-quality holograms from a single red–green–blue (RGB)-depth image via a convolutional neural network, as shown in Fig. 4A. This method exhibited a breakthrough in computational speed. Its compact CNN model occupied only 620 kB of memory, achieved real-time generation at 60 Hz for a resolution of 1,920 × 1,080 pixels on a consumer-grade GPU, and attained a speedup of over 2 orders of magnitude compared to traditional physics-based methods. In 2022, Shi et al. [63] introduced layered depth images as a data-efficient 3D representation and combined it with a novel 2-stage training protocol to achieve end-to-end synthesis of high-quality 3D phase-only holograms, further enhancing computational efficiency. The proposed method maintained real-time generation on a consumer-grade GPU and reached 5 fps on an iPhone 13 Pro, delivering improved computational speed over its predecessor. In 2025, Zhang et al. [64] proposed a symmetric dual-network framework based on residual block-based complex-valued neural (ResC-CNN) network for real-time CGH generation, reaching an average frame rate of 71.43 fps.

As a highly configurable integrated circuit, field-programmable gate arrays (FPGAs) enable large-scale integration, providing further hardware support for the rapid generation of CGHs. In 2020, An et al. [65] developed a slim-panel holographic video display method using FPGA for real-time processing, as shown in Fig. 4B. In 2023, Maruyama et al. [66] achieved real-time generation of CGH with resolutions of 1,024 × 1,024 through highly parallelized FPGA hardware design, optimized memory management, and high-speed high-definition multimedia interface (HDMI) data transmission, delivering an overall system acceleration of 7.73 times. In addition, the team of Chiba University designed high-performance computing boards based on digital signal processor (DSP) chips, GPUs, and FPGAs and developed them from holographic reconstrution-1 (HORN-1) to HORN-8 to enhance the computational efficiency, as shown in Table 1 [67–73]. Moreover, Sugie et al. [73] developed a special-purpose holography calculation board (HORN-8) based on FPGA, enabling fast generation of CGH with 10^8^ pixels, as shown in Fig. 4C. Leveraging the ultrahigh calculation performance of HORN-8, Yamamoto et al. [74] combined it with time-division multiplexing technology to achieve fast CGH calculation of a 3D object composed of 3.9 × 10^5^ points. Therefore, the high integration and flexibility of FPGA will play the key role in achieving broad applications of holographic 3D displays in the future.

**Table 1.** Comparison of performance across different HORN versions

HORN version	Processor	Point number	Calculation time
HORN-1 [67]	Integrated circuit chips	10,000	~180 s
HORN-2 [68]	DSP chips	100,000	~30 s (100 boards)
HORN-3 [69]	FPGA using hand wiring	2,500	~60 s
HORN-4 [70]	FPGA using hand wiring	415	~0.45 s
HORN-5 [71]	FPGA and printed circuit board	10,000	~0.0023 s (4 boards)
HORN-6 [72]	16 HORN-5 boards	100,000	~1.3 s
HORN-8 [73]	FPGA	1,000,000	~1.864 s

HORN, holographic reconstrution; DSP, digital signal processor; FPGA, field-programmable gate array.

### Holographic 3D display with high space–bandwidth product

The space–bandwidth product (SBP) constrains the practical application of holographic 3D display. SBP represents the multiplication of spatial information capacity and angular information capacity, which also means the product of spatial resolution and field of view (FOV). Spatial resolution determines the clarity and detail of reproduced images, limited by the pixel numbers of the SLM. The FOV defines the observation range, calculated as follows:

$$\mathrm{FOV}\approx 2\;\text{arcsin}\frac{\lambda }{2p},$$ (5)

where *λ* denotes the wavelength and *p* represents the pixel pitch of the SLM. Due to limitations in SLM pixel size and wavelength, the FOV of a single SLM is typically very small (often less than 10°). To achieve immersive experiences with a wider FOV and high resolutions, researchers have proposed various strategies to overcome the SBP limitations.

#### Holographic multiplexing technology

The multiplexing technology is one of the most typical methods for increasing SBP in holographic 3D display, which mainly includes time-division, spatial, angular, frequency, and wavelength multiplexing methods [75–79]. Within such multiplexing-based approaches, different regions or FOVs of the object are deliberately folded and encoded to partially or fully overlap within the effective FOV of the image sensor, thereby improving information utilization efficiency under hardware constraints.

Time-division multiplexing technology exploits the visual persistence effect of the human eye, using a high refresh rate SLM to sequentially display different images at distinct time intervals, thereby achieving a wide FOV through rapid angular scanning. In contrast, spatial multiplexing technology uses multiple SLMs oriented at different angles, each responsible for reconstructing a subviewing region. These subviewing regions are optically stitched together to form a continuous and expanded FOV. In practice, multiple SLMs are typically arranged in planar or circular configurations. Planar layouts offer limited FOV scalability, making circular arrangements more attractive [80]. However, circular arrangements introduce gaps between SLMs, preventing the reconstruction of a continuous object field and compromising visual quality.

Kozacki et al. [81] introduced a multiplexing approach combining space and time division by using 6 SLMs arranged in a circular configuration. This system extended the horizontal FOV from 17.5° to 35° and the vertical FOV from 1.6° to 3.3°, respectively. Liu et al. [82] further advanced this concept by integrating time-division multiplexing with pixel separation, employing 3 planar SLMs to generate subholograms with different random initial phases. This method achieved a 40 times of FOV expansion, expanding from 0.3 to 12.3 mm at a viewing distance of 600 mm. In 2025, a time-division multiplexed binocular stereoscopic holographic system leveraging human persistence of vision was proposed [83]. By using an SLM to rapidly alternate between displaying holograms for the left and right eyes, this approach achieved stereoscopic perception and provided a viable technical pathway for developing wide-viewing-angle and high-refresh-rate true 3D display effect.

#### Holographic diffraction element

**Fig. 5.**
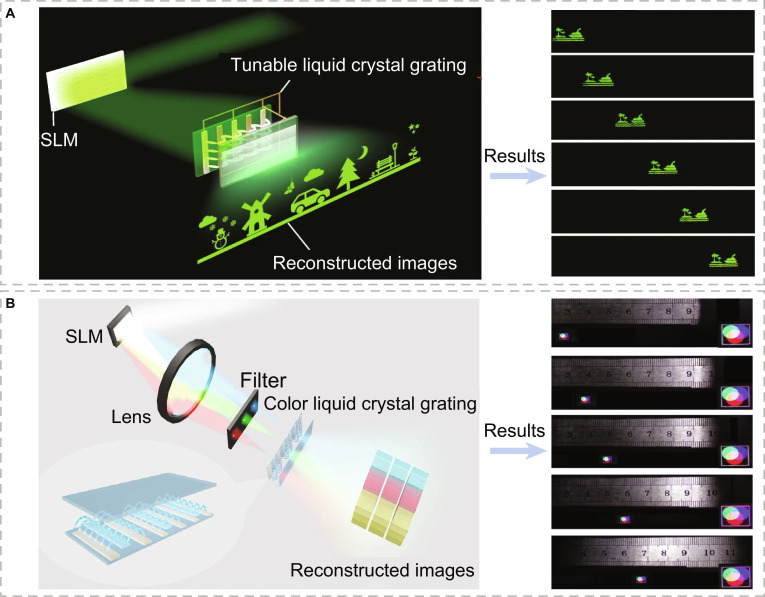
Holographic 3-dimensional (3D) displays with large field of view (FOV) based on diffraction elements. Large FOV holographic 3D display based on (A) a tunable liquid crystal grating [84] and (B) a color liquid crystal grating [85]. References [84,85] are reprinted with permission from Springer Nature.

In recent years, the emergence of new optical diffraction elements has provided a new solution for holographic 3D display with large SBP. In 2022, a holographic 3D display system based on a tunable liquid crystal grating was proposed [84], as shown in Fig. 5A. By utilizing secondary diffraction effects of the tunable liquid crystal grating, the proposed system significantly expand the FOV to 57.4°, enhancing both the size and the perspective. In 2024, a color liquid crystal grating was further designed, as shown in Fig. 5B, achieving a full-color FOV of 50.12° and effectively reducing color distortion [85]. Furthermore, an achromatic liquid crystal grating was used for large FOV and full-color holographic 3D display, achieving a breakthrough FOV of 65°, thereby enhancing display performance and marking an important advancement in holographic 3D display technology [86].

When designing holographic display systems with large FOV, holographic optical elements (HOEs) are often used. For example, placing an HOE in front of the SLM allows the limited-angle light beams emitted by the SLM to undergo secondary diffraction, greatly expanding the angle of the outgoing light. Duan et al. [87] proposed a full-color near-eye holographic display system by using a dual-layer diffraction structure in the HOE, achieving a wide FOV of 80° and an expanded eyebox, while maintaining full-color display capability. Sando et al. [88] developed a system for ultra-wide-angle holographic augmented reality with a conical HOE, achieving a horizontal FOV of 140° and a vertical FOV of 30°. Pensia et al. [89] introduced a compact off-axis digital holographic system based on a multifunctional HOE, which simultaneously performs beam deflection, focusing, and combining in a single element, increasing the FOV to 22 mm. Dashdavaa et al. [90] proposed a lensless holographic near-eye display system using divergent spherical wave illumination and multiplexed HOEs, expanding the eye movement range to 7 mm and the FOV to 25°, without compromising between the FOV and eyebox size.

In addition, the introduction of optical diffuser in the holographic reconstruction process can also expands the SBP. Yu et al. [91] used an off-the-shelf diffuser as the scattering element to increase the number of the focused point, achieving an enlargement of the SBP of holographic 3D display. Park et al. [92] proposed a flat-panel wavefront modulator to achieve holographic 3D display with large size and wide viewing angle. By using an ultrahigh-capacity nonperiodic photon sieve in conjunction with high-resolution liquid crystal display panel, the diffraction angle of the light field was significantly increased. Kuo et al. [93] used a scattering element with a thin transparent mask to improve the transmittance of light, and the proposed method achieved holographic display with an FOV of 120° and an eye box of 1 cm. In 2023, Yu et al. [94] introduced scattering medium with inherent randomness during holographic reconstruction process to suppress reconstruction interference between different depth planes, achieving crosstalk-free and multidepth holographic projection. This method significantly expands the depth of holographic reconstruction and improves SBP.

#### Metasurface-based holographic 3D display technology

With the advancement of micro and nanotechnology, metasurface, as an emerging class of optical materials, offers numerous advantages, including high resolution, wide FOV, high integration density, and multidimensional modulation capabilities [95–101]. Therefore, metasurface-based holographic 3D display technology has become one of the key technologies for holographic 3D displays with large SBP.

Researchers have expanded the modulation dimensions of metasurface to increase the number of channels in metasurface-based holographic 3D display, thereby enhancing the SBP. In 2021, Li et al. [102] introduced the off-diagonal elements of the Jones matrix as an additional degree of freedom, achieving a metasurface-based holographic display with 3 independently addressable polarization channels, which expanded the information capacity and improved the SBP. Furthermore, in 2023, Xiong et al. [103] engineered optical response noise into the Jones matrix to break the correlation between different polarization channels, realizing independent display across 11 polarization channels. By combining this approach with spatial multiplexing technology, they further extended the channel number to 36, substantially increasing the SBP. In 2025, Meng et al. [104] designed a momentum-space sparse *k*-vector filtering aperture array based on dispersion principles and integrated a Transformer neural network to optimize a wavelength–vortex multiplexed phase-only meta-hologram, achieving a holographic display with 118 channels. 

In addition to expanding the number of channels, extending the depth dimension can also effectively increase the SBP. In 2024, researchers introduced angular spectrum diffraction theory into metasurface-based holographic 3D display and imposed constraints on its transfer function, thus overcoming the depth limitation inherent in Fresnel diffraction [105]. This approach enabled a large depth reconstruction of 0.95 dm. This work provides a promising solution for realizing holographic 3D display with high SBP.

In addition to these above technologies, researchers have worked on curved CGH generation, and curved holographic technology also provides an effective technical path for achieving wide FOV and high SBP in 3D displays [106–108]. Curvature has evolved beyond a simple morphological change to become a systemic innovation that integrates geometric optics, diffraction optics, computational holography, and advanced materials. With the development of flexible optoelectronic devices, more efficient optimization algorithms, and dynamically adjustable curved surface technologies, curved holographic technology will play a crucial role in overcoming the limitations of current 3D display technologies.

### Holographic 3D display with speckle noise suppression

Holographic 3D display typically relies on laser sources due to the requirement for coherent illumination. However, the use of laser introduces the speckle noise, a granular intensity pattern superimposed on the reconstructed image. Besides, the causes of speckle noise in holographic 3D display can also be attributed to both the software and the hardware factors. The software refers to the CGH generation process, and hardware refers to the optical reconstruction process. Specifically, during the generation process, the approximate sampling is regarded as an unavoidable quantization error in the hologram. This error leads to uncontrolled coherence effects during the reconstruction process, thereby generating speckle noise. When the phase in the reconstructed image is a random distribution, the interference among object points caused by phase differences results in randomly changed intensity, significantly influencing the reconstruction quality. Therefore, how to reduce speckle noise in holographic 3D display is one of the key research directions [109–111]. Current approaches for speckle noise suppression can be broadly categorized into superposition, spatial-coherence construction, temporal-coherence destruction, and deep learning.

The superposition-based methods primarily rely on the averaging strategy from statistics. During the generation process of a series of holograms for a 3D object, the statistically independent random phases are incorporated. By using the time-multiplexing method, these holograms are sequentially displayed on an SLM within the response time of the human eyes. According to the visual transient effect of the human eye, speckle noise in the superimposed image is effectively suppressed [112]. This approach is compatible with all types of holograms. However, due to the need for multiple holograms to be refreshed, it imposes high demands on hologram generation efficiency [113]. Moreover, the time-multiplexing method consumes the refresh resources of the SLM, so a high refresh rate digital micromirror device (DMD) with the binary holograms can be used to reduce the speckle noise [114,115].

**Fig. 6.**
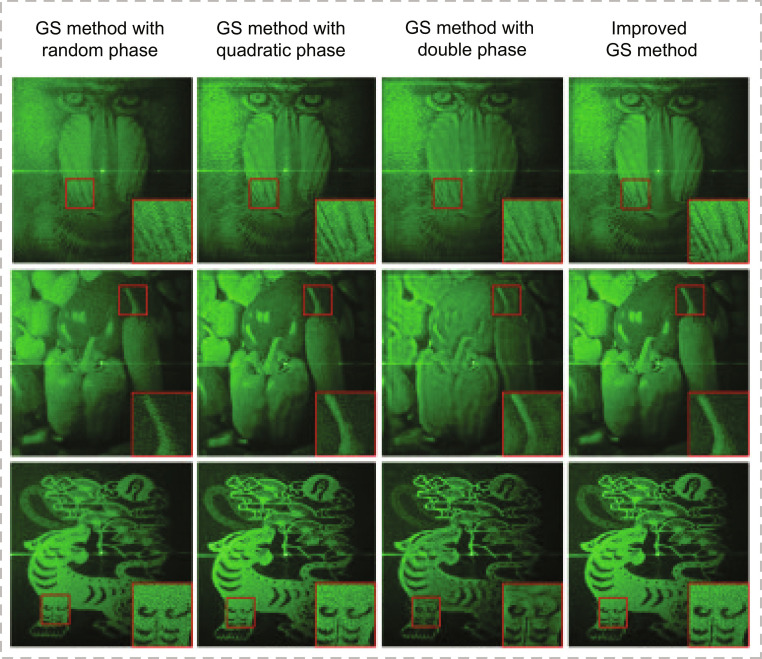
Reconstruction results of different Gerchberg–Saxton (GS) methods, including the GS method with random phase, the GS method with quadratic phase, the GS method with double phase, and the improved GS method with a dynamic compensation strategy [118]. Reference [118] is available under the Creative Commons CC-BY-NC license, 2021 Elsevier B.V.

In the hologram generation process, the complex amplitude field needs to be encoded into either a phase-only or amplitude-only hologram, and the approximate sampling is regarded as the quantization errors. These quantization errors introduce speckle noise and result in destructive spatial interference among the reconstructed image point spread functions. Therefore, to reduce the quantization errors, the spatial-coherence construction methods have been proposed to suppress the speckle noise [116]. A desired complex amplitude field can be synthesized in a phase-only or amplitude-only hologram by using double-constraint algorithm, analytic multiplexing, double-phase decomposition, double-amplitude decomposition, and stochastic gradient descent methods [117–121]. Figure 6 shows reconstruction results with suppressed speckle noise based on different GS methods [118].

The temporal-coherence destruction methods suppress speckle noise using light sources with low temporal coherence, such as superluminescent light-emitting diodes (LEDs) and micro-LEDs [122]. To further reduce the coherence, a random laser has been demonstrated usefully [123]. In conventional coherent light sources, the low spatial-coherence and high collimation are typically mutually exclusive. As a new kind of light source, the random laser is ideal because it possesses both properties. In addition, spatial filters can be used to shape the spatial coherence of a light source and render it temporally incoherent. For instance, an LED source combined with a spatial filter has been demonstrated to reduce speckle noise in holographic displays [124].

In recent years, with the advancement of artificial intelligence technology, the deep-learning-based methods can synergistically optimize real-time physical rendering for speckle noise suppression, significantly improving both the reconstruction quality and the hologram generation speed [125,126]. According to the constraints applied during the network training process, the deep-learning-based methods are divided into data-driven and physics-model-driven deep learning methods [127–129].

Shi et al. [62] combined a data-driven deep learning model with a modified double phase encoding approach to achieving high-quality real-time holographic display. The training dataset comprised 4,000 RGB-depth images generated by a random 3D scene generator, and the corresponding complex amplitude labels were obtained using a point-based method. Then, the authors further optimized the network and proposed Tensor-Holo-v2 network [63]. By using neural network preprocessing to skip the manual parameter selection in the double phase encoding, this network achieved the end-to-end training from images to the phase holograms. However, the key limitation of the data-driven approach is that the final predictions heavily depend on the choice of dataset used. Therefore, the quality of the dataset determines the upper bound of the prediction quality. In order to achieve satisfactory results, the volume of the dataset tends to be large, which makes the training process time-consuming. To overcome these limitations, physics-model-driven methods have been developed [59,60]. By using diffraction models as constraints during training process, the physics-model-driven methods can eliminate the requirement to construct datasets for phase-only holograms, thereby achieving faster generation speed and higher prediction quality. Through such training strategies, the physics-model-driven methods can learn the characteristics of the hologram with high precision, so as to eliminate speckle noise effectively.

## Application Prospect of Holographic 3D Display

Compared with the traditional 2D and stereoscopic display, a holographic 3D display can reconstruct 3D scenes with complete depth cues and provide viewers with comfortable and natural viewing experience. Therefore, a holographic 3D display offers a promising way for realistic, cost-effective, and efficient military training and simulation exercises. For example, Zebra Image Company has developed holographic 3D maps and holographic imaging systems [130]. To further develop a revolutionary holographic 3D display, the Defense Advanced Research Projects Agency launched the Urban Photonics Sandtable Display (UPSD) program to create a full-motion, full-video-rate, full-color, and interactive holographic 3D display. In recent years, the application fields of holographic 3D display have been expanding continuously. In the following sections, several key areas are introduced, including automotive head-up display (HUD), remote communication, near-eye display, and optical encryption and storage.

### Holographic HUD

**Fig. 7.**
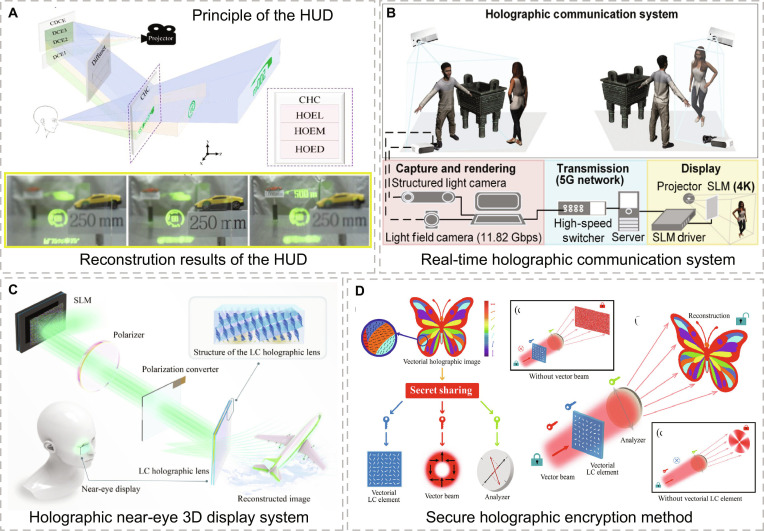
Applications of holographic 3-dimensional (3D) display. (A) Principle and reconstruction results of the holographic head-up display (HUD) [131]. CDCE, composite dispersion compensation element; DCE1, dispersion compensation element 1; CHC, composite holographic combiner; HOEL, holographic lens; HOEM, holographic mirror; HOED, holographic diffuser. (B) Real-time holographic communication system and its reconstruction results [140]. (C) Prototype of holographic near-eye display system [142]. LC, liquid crystal. (D) Concept of a secure holographic encryption method [160]. References [131,140] are available under the Creative Commons CC-BY-NC license, 2022 Elsevier B.V. References [142,160] are reprinted with permission from Copyright 1999–2026 John Wiley & Sons Inc. or related companies.

In the automotive sector, holographic HUDs can provide navigation at multiple depths that appear integrated with the real scenes. This allows the drivers to keep their eyes on the road [131,132], as shown in Fig. 7A.

Wang et al. [133] proposed a holographic augmented reality (AR) HUD system composed of volume HOEs and SLM. This system projected a 100-inch virtual image at 10-m distance, delivering equivalent brightness of >10^4^ nits with laser power consumption of <0.6 W. To further improve the driving safety, Skirnewskaja et al. [134] developed a holographic HUD system that reconstructed the Light Detection and Ranging (LiDAR) data of public roads generated by 3D terrestrial laser scanner. By combining with holographic HUD and LiDAR data of real scenes, this system allowed for identifying and alerting road obstacles that might not be easily noticed, thereby improving safety. Automotive HUD developer Envisics, which received an investment of approximately 50 million dollars from automotive giant companies in 2020, has also focused on holographic HUDs. Envisics developed a patent-protected dynamic holographic HUD based on SLM to enhance in-car user experience and cater to advanced driver assistance [135]. In 2022, SeeReal from Germany proposed a full-color, real-time 3D holographic HUD with continuous depth [136]. Combined with eye-tracking and beam steering, the proposed system allowed for the driver viewing within a large headbox of 140 mm × 70 mm.

### Holographic remote communication

Holographic communication captures people and surrounding environments at remote locations, then encoding and transmitting the holographic data over a network, and finally reconstructing dynamic 3D images of both people and surrounding environments at the terminal [137]. As holographic 3D display represents a kind of 3D display technologies, holographic remote communication is expected to provide vivid and immersive 3D communication experiences.

Yanagihara et al. [138] proposed a holographic display system capable of real-time 3D video display of real scenes with deep depth. The proposed system successfully reconstructed a moving person in the real world at 14 fps in monochrome and 5 fps in full color. In addition, VividQ, a high-profile start-up in holography, realized live holographic 3D video call through a novel pipeline named 3CPT [139]. This novel pipeline used an iPhone to capture images and depth data, used self-developed software development kit to generate, encode, transmit, and decode hologram data, and finally reconstructed with hardware. It is worth mentioning that the 3CPT pipeline only utilized widely accessible devices, thereby expanding the practical reach of holographic remote communication. He et al. [140] developed a real-time holographic communication system with a frame rate of 20 fps and a latency of 0.1 s based on a 4K SLM, offering another possibility for telepresence 3D communication. As shown in Fig. 7B, in this system, the structured light camera and light field camera were used to capture and render real 3D scenes, and holograms were then generated by the layer-based angular spectrum method and transmitted by 5G network.

### Holographic near-eye display

Among all the application scenarios for holographic displays, holographic near-eye display is perhaps the most anticipated, which is expected to provide the viewer with immersive 3D scenes that include all the depth information such as motion parallax, binocular disparity, accommodation, and vergence. Since it fundamentally avoids viewing discomfort and can inherently correct for myopic vision without additional optical components, holographic near-eye display fits a wider range of users [141]. Moreover, holographic near-eye display is regarded as a promising solution to AR, virtual reality, and mixed reality, as shown in Fig. 7C [142].

Chen and Chu [143] proposed a holographic AR display system that used a beam splitter as an optical combiner to blend the holographic images with the real world. To advance the development of holographic commercial products, researchers from Microsoft proposed a truly compact holographic near-eye display system with the appearance of eye-glasses, which had a wide FOV up to 80° [144]. In addition to a full-color, high-contrast, and speckle-free 3D display, the proposed systems also demonstrated the ability of vision correction for astigmatic vision and per-pixel focal control.

The FOV and eyebox size of the holographic near-eye display system are 2 important parameters related to viewing experience. To increase the eyebox size without sacrificing the FOV, Choi et al. [145] developed a holographic near-eye display system by taking the advantage of HOE and high-order diffractions of SLMs to realize 2D expanded eyebox size. Besides, Ooi et al. [146] used multiple beam splitters as an eyepiece and implemented dihedral corner reflector array also contributed to a lensless electroholography glass with large eyebox. To fully exploit the wavefront modulation potential of holography, Zhang et al. [147] leveraged complex amplitude modulation (CAM) to build monocular holographic near-eye system. By utilizing an Abbe–Porter filter system and a curved reflective structure, the diagonal viewing angle of the CAM-based prototype was increased to 45.2°.

In recent years, the development of new materials and advanced optical components have provided new solutions to holographic near-eye display with demanding features. Specifically, introducing random phase mask, high-aperture metalens, and compact metasurfaces can greatly increase the FOV and SBP of holographic near-eye display system without bulky optical components [148–150].

### Holographic encryption and data storage

In the current information age, information security and high-quality data storage have emerged as pivotal research directions. Due to the fact that holographic display can regulate different physical properties of light, including wavelength, polarization, and incident angle, holographic encryption and data storage are research hotspots [151,152]. In recent years, with the rapid advancement of micro and nanotechnology, metasurface has become a hot research topic [153]. Owing to its high integration density and multidimensional modulation capability, metasurface opens new avenues for advancing holographic encryption and data storage [154–157].

For instance, Zhao et al. [158] designed a birefringent vectorial metasurface, achieving 3D display with 12 crosstalk-free channels, which is capable for high-fidelity, broadband, and multichannel encryption. Qu et al. [159] demonstrated a reprogrammable all-dielectric metasurface that encrypted holographic information into dual matrices, thereby enabling arbitrary and high-capacity holographic encryption with enhanced security. Furthermore, in 2025, Xu et al. [160] proposed a secure holographic encryption method by integrating vector optical fields with a static liquid crystal device, where secret information was divided and encoded into multiple carriers, enabling multichannel encryption through dynamic control of incidence polarization and analyzer orientation, as shown in Fig. 7D. In addition, by introducing polarization modulation, wavelength modulation, and inverse design, researchers are committed to further expanding the number of channels for metasurface-based holographic display, thus providing possibilities for high-capacity holographic data storage [161–163].

## Conclusion

**Fig. 8.**
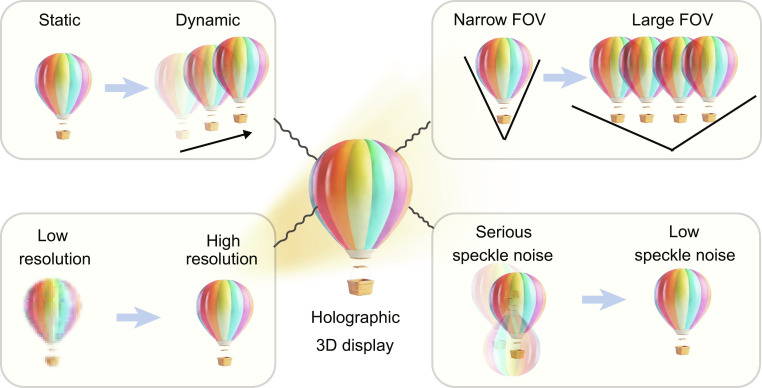
Development of the holographic 3-dimensional (3D) display.

Holographic 3D display technology, capable of recording and reconstructing the complete wavefront information of 3D objects, has become one of the most ideal approaches for realizing ideal 3D display. Over the years, holographic 3D display technology has undergone a series of transformations, from static to dynamic, from low resolution to high resolution, from narrow viewing angle to wide viewing angle, and from serious speckle noise to low speckle noise, as shown in Fig. 8, continuously evolving toward the ideal 3D display and holding promise for transitioning from laboratory prototypes to commercial applications.

In this paper, we have focused on the technical bottlenecks of holographic 3D display and conducted a detailed summary and analysis of its key technologies and future application developments. In terms of fast generation of CGHs, the synergistic integration of hardware computational power and artificial intelligence may be the key to achieving real-time holographic 3D display. Traditional CGH generation methods can be categorized into point-based, polygon-based, and layer-based approaches depending on the presentation ways for 3D objects. These methods optimize the mathematical representation, but the calculation complexity continues to be constrained by the complexity of the 3D object, making real-time generation of CGHs for complexed objects difficult. With advances in computer science, the leap in hardware performance offers a more direct and noticeable improvement in CGH generation speed. More importantly, the flourishing development of artificial intelligence has enabled deep learning to play an increasingly important role in accelerating hologram generation and inverse design. However, the substantial computational and memory demands of deep learning models, particularly for high-resolution and large-depth scenes, may still exceed the capacity of current hardware. In addition, as network performance heavily depends on the representativeness and diversity of the training dataset, it is difficult to ensure the generalization across diverse and dynamic 3D scenes. Therefore, in the future, deep integration of high-performance calculation hardware with advanced deep learning models is expected to achieve real-time holographic 3D display.

In terms of holographic 3D display with high SBP, optical component innovation will be the core driver. Currently, although holographic multiplexing technology and holographic diffraction elements can both effectively expand the viewing angle of holographic reconstructed images, they are ultimately limited by the resolution and diffraction angle of the devices themselves. As a novel type of optical material, metasurface featuring high resolution and large diffraction angle offers a new enabling solution for high SBP holographic 3D display technology. However, the design of metasurface structure is highly complex, particularly when simultaneously modulating multidimensional information such as phase, amplitude, and polarization, making it difficult to ensure structural stability. In addition, the dynamic modulation capability of metasurface is constrained by the response speed of the constituent materials, posing challenges to the realization of flexibly reconfigurable metasurface-based holographic 3D displays. Furthermore, the high fabrication cost of metasurface hinders its transition toward practical applications. In the future, with continuous advances in inverse design algorithms, tunable materials, and nanofabrication technologies, metasurfaces hold promise to address the fundamental limitations of high-SBP holographic 3D display technology.

In terms of high-quality holographic 3D display, deep learning methods will dominate future speckle noise suppression technologies. To address the persistent problem of speckle noise in holographic 3D display, deep learning methods have demonstrated potential beyond traditional optical suppression technologies. Leveraging deep learning for wavefront optimization and image enhancement will be a critical pathway to achieving high fidelity, low speckle noise, and high-quality holographic 3D display. However, current deep learning models often lack interpretability and generalization across varying optical setups, and the acquisition of large-scale, high-quality training datasets with ground-truth holograms remains difficult and time-consuming, which may hinder model performance and scalability. In the future, integrating physics-informed neural networks to combine data-driven learning with physical constraints may improve model interpretability and generalization, thereby enabling high-quality holographic 3D display.

Due to its ability to fully record and reconstruct the complete wavefront information of 3D objects, holographic 3D display holds broad application fields, such as automotive HUDs, remote communication, holographic near-eye displays, and optical encryption and data storage. Among these, holographic near-eye display is widely recognized as one of the most promising directions and is likely to be among the first to achieve widespread adoption. As holographic 3D display technology continues to develop and optimize, its application potential is being further explored. With the rapid development of micro- and nanofabrication, metasurface-based holographic 3D display technology will show greater application prospects in many frontier fields such as holographic communication and holographic storage.
